# Armed Rollers: Does Nestling’s Vomit Function as a Defence against Predators?

**DOI:** 10.1371/journal.pone.0068862

**Published:** 2013-07-10

**Authors:** Deseada Parejo, Jesús M. Avilés, Aránzazu Peña, Lourdes Sánchez, Francisca Ruano, Carmen Zamora-Muñoz, Manuel Martín-Vivaldi

**Affiliations:** 1 Estación Experimental de Zonas Áridas (EEZA-CSIC), Ctra. de Sacramento s/n, La Cañada de San Urbano, Almería, Spain; 2 IACT (CSIC-UGR), Avda. de las Palmeras, 4, Armilla, Granada, Spain; 3 Estación Experimental del Zaidín (EEZ-CSIC), Profesor Albareda, 1, Granada, Spain; 4 Departamento de Biología Animal y Ecología, Facultad de Ciencias, Universidad de Granada, Campus Universitario de Fuentenueva, Granada, Spain; Universidade de São Paulo, Faculdade de Filosofia Ciências e Letras de Ribeirão Preto, Brazil

## Abstract

Chemical defences against predators are widespread in the animal kingdom although have been seldom reported in birds. Here, we investigate the possibility that the orange liquid that nestlings of an insectivorous bird, the Eurasian roller (*Coracias garrulus*), expel when scared at their nests acts as a chemical defence against predators. We studied the diet of nestling rollers and vomit origin, its chemical composition and deterrent effect on a mammal generalist predator. We also hypothesized that nestling rollers, as their main prey (i.e. grasshoppers) do from plants, could sequester chemicals from their prey for their use. Grasshoppers, that also regurgitate when facing to a threat, store the harmful substances used by plants to defend themselves against herbivores. We found that nestling rollers only vomit after being grasped and moved. The production of vomit depended on food consumption and the vomit contained two deterrent chemicals (hydroxycinnamic and hydroxybenzoic acids) stored by grasshoppers and used by plants to diminish herbivory, suggesting that they originate from the rollers’ prey. Finally, we showed for the first time that the oral secretion of a vertebrate had a deterrent effect on a model predator because vomit of nestling rollers made meat distasteful to dogs. These results support the idea that the vomit of nestling rollers is a chemical defence against predators.

## Introduction

Chemical defence is one of the mechanisms that organisms use to enhance their survival prospects. Several different animal taxa from arthropods [1]–[3] to amphibians [4] defend themselves against predators, parasites and/or competitors by producing deterrent or noxious substances [5]. Within amphibians, poison frogs that sequester alkaloids from their prey to be chemically protected are the best known example [4]. In other groups, such as endothermal tetrapods, however, examples of chemically defended animals are limited to a small number of lineages [6]. Indeed, birds have not been included in lists of chemically-defended animals until the end of the twentieth century [5], when species of the genus *Pitohui* were found to deter predators and/or parasites by means of the neurotoxin homobatrachotoxin that is contained in their tissues [7]. Apart from this extreme example of poisonous *Pitohuis*, several other bird species also contain toxic or unpalatable compounds that may help to defend them (see reviews in [5], [8]). For instance, hoopoes *Upupa epops* and green woodhoopoes *Phoeniculus purpureus*, when disturbed at the holes where they roost, secrete drops of a fetid substance from their uropygial glands [9].

The full understanding of the functioning of a chemical defence needs to address the three following general issues [5]: a) the origin of defensive chemicals, which can either be produced *de novo* by the defended organism or be obtained from other organisms, usually through consumed food; b) the composition of the defensive substances; and, c) the effects of the chemicals on generalist predators because chemical defences are effective against generalist consumers but can be circumvented by specialists [10]. This research pathway has been widely adopted to investigate the evolution of chemical defences in several taxa [4], [11], [12]. However, many of the examples of avian chemical defence are based on anecdotal reports [5]. Therefore, the ecological and evolutionary relevance of avian chemical defences still needs to be thoroughly investigated.

Here, we aim to investigate in depth the possibility that the odorous orange substance that Eurasian roller (*Coracias garrulus*) nestlings regurgitate when disturbed [13] acts as a defence. Vomiting is a particular behaviour of the nestlings of rollers, and, as far as we know among birds, only described in chicks of some Procellariiform species [14]. Roller parents returning from a foraging trip approach to their nests more cautiously when they smell nestling vomit, suggesting they have interpreted this smell as a signal of offspring fear [13]. However, the primary function of this substance is unknown. The expelling of vomit must be costly for nestlings due to the loss of body fluids; thus, it seems unlikely that the secretion has no function at all. Indeed, as indicated above, this vomiting behaviour is not a common feature of nestling birds but limited to a small number of species. Consequently, we hypothesize that the vomit might have a defensive function in rollers by making nestlings distasteful to predators.

Many herbivorous insects such as grasshoppers regurgitate when disturbed [15]. The defensive role of the expelled fluid has been attributed primarily to ingested plant secondary compounds [16]–[18]. Grasshoppers are the main prey that rollers hunt to feed their nestlings ([19], see below. Furthermore, rollers feed their offspring with a large share of poisonous arthropods [19] that are avoided by most of the other sympatric insectivorous birds [20]. This suggests that rollers are resistant to these toxic substances and could have the ability to sequester chemicals from their protected prey to defend themselves, like phytophagous insects do with plants secondary compounds [15], [21], [22]. Therefore, we expected that vomit expelled by nestling rollers contained the defensive substances used by plants as anti-herbivore defence, but accumulated in some specialized herbivores that are able to circumvent this defensive strategy as the phytophagous arthropods consumed by rollers. The effect that it produces on potential predators is crucial for assigning a defensive role to the substance. Indeed, if vomit has a defensive function, it should be produced in response to a threat and elicit rejection or avoidance by predators, parasites and/or microbes.

In this context, we combine detailed diet and behavioural analysis of roller nestlings, experimental approaches and chemical analyses with high performance liquid chromatography–mass spectrometry (LC-MS) to address the following four objectives: 1) whether the substance that nestling rollers expel has a dietary origin; 2) the type of stimulus (mobile, visual, auditive or tactile) that triggers its expelling; 3) the composition of nestling oral secretion; and, finally 4) whether it is effective eliciting aversion by generalist predators.

## Methods

### Study System and Sample Collection

The roller is a migratory socially monogamous bird that lays one clutch per year of about 5 eggs (mean ± s.e. = 5.20±0.12, N = 60 nests in the study period).

The study was carried out from mid-May to July of 2008 to 2012 in a nest-box breeding population in south-eastern Spain (37°18′N, 3°11′W) (see [23] for details). During the study years all reproductive events were precisely monitored (laying date, clutch size and incubation time) in order to estimate hatching date. Once the first nestling hatched in each nest we visited them daily during the hatching period to record the age and/or size of nestlings when they begin to produce vomit. Later throughout the nesting period, we collected vomit samples every fourth day. The samples from each nestling were collected separately in 2 ml vials and stored refrigerated at about 5°C until being frozen within the same day (up to 6 h later) in the laboratory. Some of the samples were used for the analysis of chemical composition and others for deterrence tests with dogs.

### Nestling Diet

From 2008 to 2011 we collected data on nestling diet by identifying prey (up to order level) offered by parents to nestlings from video recordings. In all years we recorded parental provisioning behaviour at nests with 10 day-old nestlings and in 2008 and 2009 also with 18 day-old nestlings.

### Behavioral Study: Stimulus Inducing Vomit

In 2011 and 2012, we recorded whether each individual nestling vomited or not and weighed them to later associate with age and size of nestlings with vomit production. At each nest we wrote down the type of stimulus that induced vomiting. For that purpose when we arrived to a nest, we opened the nestbox and then followed the next sequence of actions: 1) to speak loudly to nestlings, 2) to show our face to them, 3) to gently touch them, and, finally, 4) to take them in the hand one by one and gently shake them. Actions were separated by ten-second periods. This sequence of actions allowed us to test whether vomiting was in response to an auditive, visual, tactile or mobile stimulus.

### Food Deprivation Experiment: Vomit Origin

In 2012, we performed an experiment using neck collars to deprive nestlings of food and thus test food as the source for vomit production. At each nest with 7 to 20 day-old nestlings (age at which the vomit is expelled, see below), we took all nestlings and assigned them randomly to one of the following two treatments: with or without neck collar. Collars were gently applied to the neck of chicks in such a way that they prevented the transit of prey to the bird’s digestive while allowing birds to breath and expel out vomit. We are certain that collars do not restrict nestlings’ ability to vomit because none of the nestlings that stopped vomiting after collar application vomited after collar removal. Furthermore, many of the nestlings that vomited at the beginning of the experiment reduced their vomit production after collar application but still continued on vomiting. This approach has been widely used to study the diet of insectivorous birds and proved to be innocuous for nestlings [19], [24]. Before and after the experiment, we weighed each nestling and estimated the amount of vomit they produced: (a) normal production, when the vomit overflowed from the beak and fell down abundantly; (b) medium production, when only some drops of vomit fell down from the beak; (c) scarce production, when vomit did not overflow the beak and only could be seen into the oral cavity; and (d) no production of vomit. After 1 hour, neck collars were removed and prey in nestlings’ oral cavity were collected and stored in ethanol until their identification. No nestling increased its production of vomit after the experiment (probably due to the fact that the manipulation stimulated them to vomit twice in an hour and the production/expulsion of vomit is likely to be costly for nestlings). Hence, we used the decrease in vomit production (decrease *versus* non-decrease/maintenance of vomit production) as the response variable to the experiment. We expected a decrease in vomit production in nestlings with neck collars if the origin of the oral secretion was food and not glandular.

### Chemical Analysis: Vomit Composition

We restricted our analyses to the following compounds that were known to be present in chemically defended plants against herbivorous arthropods or in chemically defended arthropods against predators: L-hyoscyamine [25], [26], Psoralen and Bergapten [17], Hydroxycinnamic acid and Hydroxybenzoic acid [27]–[30], Benzoquinone [12], [31], [32] and Dihydronepetalactone [2], [33].

#### Method of extraction and analysis

A sample of homogenised vomit (100 µL) was measured with an automatic pipette and passed to a 15-mm glass tube to which 2.5 mL MilliQ water and 240 µL glacial acetic acid was added. The sample was stabilized for 5 min, added with 2.5 mL diethyl ether and vortexed at the highest velocity for 1 min. The mixture was then centrifuged (4000 rpm, 5°C, 5 min) and the organic layer transferred to another tube. The remaining aqueous phase was extracted twice again with 2.5 mL diethyl ether. The combined organic phases were evaporated in RapidVap (Speed: 76, 60°C, 4 min) to almost dryness and then to dryness under a gentle stream of N_2_ (20–30 min approximately). The dried extract was dissolved in 150 µL of acetonitrile (LC MS Grade, Fisher): MilliQ water before injection.

The samples were analysed using a HPLC separation module (Allience 2695, Waters) with a Quattro Micro triple quadrupole mass spectrometer detector (Waters, Milford, MA). Instrument control, data collection, analysis, and management were controlled by MassLynx 4.0 and Quanlynx V4.1 software packages. Separation was performed using an Atlantis T3 column (2.1×100 mm, 3 µm, Waters) connected to an Atlantis precolumn (2.1×10 mm, 3 µm, Waters) with a flow of 0.3 mL/min. The mobile phase consisted in acetonitrile and MilliQ water, both added with formic acid at 0.1%. The gradient started at 30% of acetonitrile, changed to 40% in 4 min and then changed to 30% of acetonitrile in 6 min and these conditions were held for 6 min. Retention times of the compounds are shown in Appendix S1.

The effluents from the HPLC were introduced into the mass spectrometer using an orthogonal Z-spray electrospray interface (Micromass, Manchester, U.K.). The ionization source temperature was 120°C and the desolvatation gas temperature 350°C. The cone gas and desolvation gas-flow rates were 600 and 0 L/h, respectively. The capillary voltage was 3.0 kV and the cone voltage 15 V. Argon gas (2.83 10^−3^ mbar) was in the collision cell. We optimized the mass spectrometric parameters by continuous infusion of individual solutions of each compound at 10 ppm in methanol:water (1∶1). Detection of the compounds was performed in the positive and negative ionization modes. The quantification of the compounds was based on appropriate Multiple Reaction Monitoring of ion pairs (Appendix S1).

#### Assessment of the analytical parameters

Calibration plots were constructed at two different concentration ranges (high and low) (Appendix S2). Sensitivity (smallest variation in concentration discerned), linearity, limit of detection and limit of quantification were calculated as reported in [34]. Definitions and calculations of repeatability and recovery are detailed in Appendix S2.

### Bioassay to Test for Deterrent Activity of Vomit

We assessed the deterrent effects of vomit to predators in July 2010 using dogs *Canis lupus familiaris* as the model predator. Dogs are carnivorous domestic mammals that are able to consume large meals rapidly (a legacy of competitive feeding in the wolf) and select food mainly by olfaction. Their taste system is based on what is probably a general carnivore pattern [35]. Whether the substance expelled out by nestling rollers is repulsive for generalist carnivorous mammals such as dogs, it could has also deterrent effects for wild predators. Dogs used for the experiment were temporally living in charity shelters after abandonment by their owners. These animals are regularly fed once a day, around midday, with commercial food and water. Therefore, they showed great appetite for meat. We used 3×2 cm pieces of uncooked chicken meat that were uniformly smeared with 80 µL of distilled water (control) or fresh vomit on the non-visible side (down), therefore differences in preference by one of the two pieces of meat could be only attributed to their taste and/or odour.

Before the daily feeding, two Petri dishes (50 cm apart), one containing chicken meat smeared with water and the other one containing chicken smeared with roller nestling vomit, were presented to dogs in isolation. We balanced the side (right or left) where each treatment was located across trials. Each dog and vomit was tested only once. Vomit samples used in the experiments came from different nests. Dogs’ behaviour was observed until they ate both pieces of meat or a maximum time of 10 minutes. After that time we considered dogs were non-responsive to the test. From a vantage point we recorded the option each tested dog ate first as a measure of the interest for the stimuli. In addition, we recorded whether each dog ate or not the meat smeared with vomit during the observation period irrespective of which option was taken the first and the time spent to do so.

### Ethic Statement

This study was conducted under licenses of the Junta de Andalucía (Spain) to make the fieldwork with rollers and the Ayuntamiento de Almería (Spain) to perform tests of deterrence of vomit to dogs. Hence, all necessary permits were obtained for the study, which complied the national legislation of Spain concerning animal handling. Study areas are privately owned and permission to use the areas was acquired from the land owners.

### Statistical Analyses

We performed a General Linear Mixed Model (MIXED SAS procedure) to test for the effects of the neck collar experiment on nestling weight variation. The effect of the experiment of neck collars on vomit production (decrease *versus* maintenance of vomit production) was analysed by using a Generalized Lineal Mixed Model (GLIMMIX SAS procedure). As we used all nestlings from each brood in the experiment, in both models the nest was introduced as a random factor to control for the non-independence of data from siblings.

We used a Chi-squared goodness of fit test (FREQ SAS procedure) to compare the observed frequencies in the deterrence test with dogs with the expected frequencies under a scenario of random distribution of choices (i.e. 50% prefer meat with vomit and 50% prefer meat with water).

## Results

### Nestling Diet

We identified at least one prey item provided by parents in 34 video recordings (36.2% of total recordings) from 32 different nests (50% of the observed nests). From these 34 video recordings, we identified 112 items, all of them arthropods, mainly belonging to the order Orthoptera (N = 103, 92%), but also some Coleoptera (N = 2, 1.8%), Lepidoptera (N = 3, 2.7%) and centipedes Scolopendromorpha (N = 4, 3.6%) (Table 1).

**Table 1:** Number of prey by taxa and percentage of frequency of each order (in brackets) in the diet of nestling rollers estimated from video recordings (N = 32 nests) or by the application of collars to nestlings’ necks (N = 14 nest).

	Source of prey identification	
Prey type	Video recordings	Neck collars
*O. O. Orthoptera*	103 (92.0%)	19 (90.5%)
*- F. Acrididae*		- 15 (15 *Callyptamus wattenwylianus)*
*- F. Tettigonidae*		- 4 (2 *Tettigonia viridissima*, 2 *Platycleis sp.)*
*O. Coleoptera*	2 (1.8%)	1 (4.8%)
*- F. Cetoniidae*		- 1 (*Protaeia morio*)
*O. Scolopendromorpha*	4 (3.6%)	1 (4.8%) (*Scolopendra cingulata*)
*O. Lepidoptera*	3 (2.7%)	0
**Total number of identified prey**	**112**	**21**

When species identification was possible the latin name of the species is specified in brackets.

We also collected some prey items from neck collars sporadically applied to nestlings in 2008 and in the experiment of food deprivation in 2012. Specifically, we collected 21 arthropods from 14 different nests, 19 belonged to the order Orthoptera (90.5%), 1 to the order Coleoptera (4.8%) and 1 to the order Scolopendromorpha (4.8%) (Table 1).

### Stimulus Inducing Vomit

All nestlings (N = 43) expelled out the vomit when they were moved but not in response to the other stimuli (auditive, visual or tactile). Furthermore, most nestlings began to vomit when they still were blind, indicating that at that age regurgitation cannot be a response to a visual stimulus.

The vomiting behaviour was initiated when nestlings were 6.7±0.7 days old (mean ± s.e., N = 43 chicks from 11 nests) and weighed 57.2±6.8 g (mean ± s.e., N = 34 chicks from 9 nests). Nestlings lost this behaviour when they were 19.6±0.4 days old (mean ± s.e., N = 37 chicks from 11 nests), which is around fledging time.

### Vomit Origin

In 2012 we applied neck collars to half of the nestlings (14 nestlings) from 9 nests. Collars were efficient because nestlings with neck collars lost more weight than nestlings without neck collars (General Lineal Mixed Model: F_1,18_ = 8.33, P = 0.0098. Mean weight loss = 4 g (N = 14 nestlings with collars) *versus* 0.18 g (N = 14 nestlings without collars)). Change in vomit production (i. e. decrease *versus* no decrease) varied in response to the application of collars (F_1,18_ = 11.65, P = 0.003), so that most nestlings with neck collars reduced their production of vomit (12 out of 14 nestlings), while nestlings without neck collars did not (4 out of 14 nestlings).

### Vomit Composition

The results show that all the vomit samples contained Hydroxybenzoic and Hydroxycinnamic acids although in 2 and 4 cases respectively out of 16 samples, there were only traces of the chemicals. Hydroxybenzoic acidconcentration was 481.2±77.6 ppb (mean ± s.e.) (min–max = 130.1–1139.1 (N = 14)). The content in Hydroxycinnamic acid was 150.0±26.1 ppb (mean ± s.e.) (min–max = 60.2–354.4 (N = 12)). In one sample Psoralen was found close to the Limit of Quantification (17.99 ppb) and in some samples (4 out of 16) traces of Psoralen were detected but could not be quantified since their amount was close to the Limit of Detection, but below the Limit of Quantification (<9 and >3 ppb). On the other hand, traces of Dihydronepetalactone, close to or well below the Limit of Detection (<10 ppb), were sporadically detected. Additionally, if Hyoscyamine was present it could not be detected as above indicated. Another processing system was also assayed, by using Ostro cartridges (Waters, Mildford), but the compound could not be recovered from any of the spiked vomit samples. P-Benzoquinone was also included as a candidate compound. However, this chemical showed a high resistance to be broken and did not produce any fragment under the MS conditions used here, preventing its determination with the mass spectrometric detector. The analysis by HPLC-UV at 290 nm did not lead to any positive conclusion either.

### Deterrent Activity of Vomit

We performed the deterrence test to 25 dogs, 5 of which were not responsive. Before deciding whether eating or not the offered meat, dogs either smelt (most cases) or licked it. Most of the reactive dogs (18 out of 20) preferred as the first option the meat smeared with water instead of the meat smeared with roller vomit (Goodness of fit test: χ^2^_1_ = 12.8, P = 0.0003). 12 out of 18 dogs (67%) that chose meat with water as the first option also consumed meat with vomit as the second option but they did that after 2 minutes in average (mean = 118.4 seconds). The remaining 6 dogs out of 18 only ate meat with water. Meanwhile, the 2 dogs that chose meat with vomit as the first option also ate the meat with water immediately after (mean = 31.0 seconds).

## Discussion

In this paper we first show that arthropods from the order Orthoptera are the main prey of roller nestlings in the study area. We also demonstrate that the vomit expelled by roller nestlings depends on food provided by parents and that vomiting is triggered by grasping and moving of nestlings. In addition, we have found that vomit samples contain variable concentrations of hydroxycinnamic and hidroxybenzoic acids, two phenolic acids, and that some of the vomit samples also have traces of psoralen, a furanocoumarin. Finally, we have shown that vomit of nestling rollers alone makes chicken meat unappealing for dogs. Below, we will critically assess these findings in the light of the hypothesis that nestling rollers regurgitate when disturbed, expelling an orange and odorous substance [13], which could result from the sequestration of chemicals from their prey for their own use, as their main prey (grasshoppers and beetles) do from plants. As this behaviour is produced in response to a threat, it could have a defensive function during the nesting period in which nestlings are not able to escape from predators.

We have found that the movement of nestlings by the investigator seemed to trigger vomit ejection. This fact suggests that the vomit might be produced in response to some kind of predators that actively grasp and move prey during the predation event such as snakes, rats and mustelids, which are common predators of hole-nesting species [36], [37] as rollers. Holding nestlings was always the last tested stimulus in our experiment which raises the possibility that was order, rather than stimulus *per se*, which was determining the found pattern. However, this is unlikely because we previously knew from our long-term monitoring of rollers that chicks vomited when straightly handled (Parejo and Avilés unpublished data). Anyway, which is important here is that disturbance causes vomiting.

Our results also indicate that the production of vomit depends directly on recently consumed food because when nestlings were food-deprived for 1 hour they reduced vomit production. This result suggests that the vomit has not an endogenous (i.e. glandular) but a dietary origin. The oral emissions of arthropods contain a blend of digestive enzymes, salivary secretions, and partially digested food as plant secondary compounds [15]. Therefore, in rollers a similar mechanism of secretion production seems to be feasible. Moreover, toxins seem not to be produced *de novo* by any vertebrate group [38], which leads us to think that all the chemicals found in vomit samples have a dietary origin. What is clear is that nestling rollers need food either as a source of chemicals or as a source of energy to produce the vomit.

In the study area roller nestlings are mainly fed with Orthoptera, which are relatively polyphagous species [39], [40]. Therefore, we expected to find a tritrophic effect of plant secondary compounds, from plant to insects and then to rollers. In agreement with our expectation, we found that all vomit samples contained hydroxycinnamic and hydroxybenzoic acids, that are phenolic acids usually found in leaves of many *Gramineae* and cell walls of most higher plants [18], [28], [41] and that deter insect feeding [29], [42]. Furthermore, some samples also contained traces of psoralen that is a furacoumarin produced by a wide variety of plants in response to pathogens and/or herbivore attacks [17]. Despite the occurrence of these substances in several plants, most phytophagous insects develop the ability to cope in greater or lesser extent with these unpalatable substances (see [30] for a revision). Therefore, phytophagous insects may first feed on defended plants and, second, use opportunistically plant secondary compounds for their own defence [16], [21]. Indeed, oral secretions produced by several species of grasshoppers, such as *Romalea microptera*, *R. guttata* and *Taeniopoda eques*, are dominated by phenolics and quinones [31], [32]. These armed insects, hunted by adult rollers to feed their offspring, would be the putative source of phenolic acids contained in the vomit of roller. Previous work has demonstrated that oral secretion of different grasshopper species can deter predators [16]–[18], [31]. Here, for the first time, we show the deterrent effect of the oral secretion of a vertebrate, the avoidance of the oral secretion of nestling rollers by domestic dogs. These results suggest that vomit can be used by rollers as a way to be chemically-defended, which would improve brood survival and, consequently, parental fitness. As nestlings only vomit after being grasped and moved, some of the common predators of hole-nesting birds, as rats and mustelids, must perhaps bite a nestling roller before realising that the prey is unpleasant. Thus, one could wonder about the nestling advantage of this defence. Kin selection is a possible answer to that question because a predator that finds the first nestling of a brood of five to be distasteful may leave alive the others [5]. Alternatively, the advantage might be found in parental fitness because parents would benefit from an incomplete predation event at their nest. For other predators as snakes, however, the advantage of the defence is easier to understand. Snakes would first try to immobilize nestlings by constriction while holding them with the mouth, which would induce nestling vomiting and hence the immediate savouring of the unpleasant prey through the snake olfactory tongue [37], thus avoiding chick death.

It should be acknowledged here that despite the initial avoidance that dogs showed against meat with vomit, many dogs finally ate it. However, they did that after some minutes, perhaps after the volatilization of much of the smell of the vomit [13]. This fact probably means that vomiting only serve in the short time against predators because of the volatile nature of the expelled substance. Nevertheless, it is interesting to highlight that 30% of the tested dogs avoided consumption of the meat experimentally smeared with vomit even as a second option. This result clearly shows that roller vomit can be effective in avoiding nestling predation. A direct test of predator avoidance function with natural predators would require experimental manipulation of vomit production in nests in the field and estimating its effects on predation rates. Such a protocol, however, needs the development of a method to inhibit vomiting.

To summarize, several lines of evidence support the idea that the vomit of nestling rollers might have a defensive function against predation: 1) It is expelled in response to a threat, our handling, at nests. 2) Vomit seems not to be produced *de novo* by nestlings but has a dietary origin, which suggests that vomiting might be a costly behaviour that should have an adaptive function. 3) Vomit contains deterrent chemicals used by plants against herbivores and by phytophagous insects against their predators. Therefore, these substances could be acquired by rollers from plants through prey insects to deter predation at nests. 4) The fact that the vomit makes meat unpalatable to mammalian generalist predators supports the idea that secondary compounds of plants present in the vomit could be used by rollers as a chemical defence. However, we have no data yet to show that individuals with less protection experience reduced fitness [5].

## Supporting Information

Appendix S1 Retention time (t_R_) and optimised mass spectrometric parameters for the detection of the compounds under study.
(DOCX)

Appendix S2 Assessment of the analytical parameters in chemical analyses.
(DOCX)
