# Supplementary material for: Armed Rollers: Does Nestling’s Vomit Function as a Defence against Predators?
Source: PLoS One. 2013 Jul 10;8(7):e68862. doi: 10.1371/journal.pone.0068862 (PMC3707886; doi:10.1371/journal.pone.0068862)
Supplement: Appendix S1 — Retention time (tR) and optimised mass spectrometric parameters for the detection of the compounds under study. (DOCX) [file pone.0068862.s001.docx]

Appendix S1. Retention time (t_R_) and optimised mass spectrometric parameters for the detection of the compounds under study. The quantification of the compounds was based on appropriate Multiple Reaction Monitoring (MRM) of ion pairs. Hy: L-hyoscyamine; HBA: Hydroxybenzoic acid; HCA: Hydroxycinnamic acid; Ps: Psoralen; 8M: Dihydronepetalactone; Bg: Bergapten.

| **Compound** | **t_R_ (min)** | **Ionization Mode** | **Cone (V)** | **Collision Energy (eV)** | **MRM** |
| --- | --- | --- | --- | --- | --- |
| Hy | 1.2 | ES+ | 40 | 25 | 290.30 → 124.23 |
| HBA | 1.8 | ES- | 30 | 15 | 137.00 → 93.05 |
| HCA | 2.2 | ES- | 25 | 15 | 163.10 → 118.96 |
| Ps | 6.8 | ES+ | 40 | 25 | 187.00 → 131.16 |
| 8M | 7.4 | ES+ | 40 | 20 | 217.17 → 161.10 |
| Bg | 9.0 | ES+ | 40 | 20 | 217.17 → 202.03 |
