# Supplementary material for: Armed Rollers: Does Nestling’s Vomit Function as a Defence against Predators?
Source: PLoS One. 2013 Jul 10;8(7):e68862. doi: 10.1371/journal.pone.0068862 (PMC3707886; doi:10.1371/journal.pone.0068862)
Supplement: Appendix S2 — Assessment of the analytical parameters in chemical analyses. (DOCX) [file pone.0068862.s002.docx]

**Appendix S2**. Assessment of the analytical parameters in chemical analyses.

Sensitivity (smallest variation in concentration discerned), linearity, limit of detection (LOD) and limit of quantification (LOQ) were calculated as reported in [38]. Repeatability was defined as the relative standard deviation of non-consecutive repeated measurements (N = 4) of a standard solution at high and low concentration levels (ca. 270 and 40 ppb) in the same analytical set. Reproducibility was expressed as above but concerning injections in different days (N = 4). Recovery was calculated by standard addition to a vomit sample. First, a blank was run by extracting a vomit aliquot and injecting it per triplicate. The initial concentration of each compound was determined. Other vomit aliquots from the same sample were added with 5 μL of the corresponding standard solution resulting for each compound in three concentration levels, which ranged from 33 to 333 ppb for Ps, 8M, Bg and Hy, from 133 to 333 ppb for HCA and from 233 to 333 ppb for HBA.

The analytical method had a different sensitivity for the analysed compounds (see table below). The calibration curve at the low concentration range for 8M and HBA could not be used since the response was not linear. For both compounds only the calibration curve for the higher range was employed. Recovery of the spiked samples was higher for the highest concentration level for all the compounds. It ranged from medium-high (61.7-70%) for Ps, 8M and Bg to medium-low (35.2-48.2%) for HBA and HCA. Although the analytical method for Hy was linear and very sensitive, the compound was completely lost during sample processing.

Quality parameters of the chromatographic method and efficiency of recovery from standard addition. Ps: Psoralen; 8M: Dihydronepetalactone; Bg: Bergapten; Hy: L-hyoscyamine; HBA: Hydroxybenzoic acid; HCA: Hydroxycinnamic acid

|  |  | **Analysed compounds** | | | | | |
| --- | --- | --- | --- | --- | --- | --- | --- |
| **Analytical parameters** | **Ranges** | Ps | 8M | Bg | Hy | HBA | HCA |
| Calibration conc. range (ppb) | High | 100-333 | 100-333 | 100-333 | 100-333 | 100-333 | 100-333 |
|  | Low | 2-10 | 2-10 | 2-10 | 2-10 | 2-10 | 2-10 |
| Sensitivity (ppb) | High | 5.2 | 3.2 | 4.2 | 1.5 | 7.0 | 6.4 |
|  | Low | 0.9 | - | 0.3 | 0.9 | - | 1.5 |
| Linearity | High | 0.97 | 0.98 | 0.98 | 0.99 | 0.96 | 0.96 |
|  | Low | 0.85 | - | 0.94 | 0.84 | - | 0.74 |
| LOD (ppb) | High | 16.3 | 10.2 | 13.1 | 4.6 | 21.9 | 20.2 |
|  | Low | 3 | - | 1 | 3 | - | 5 |
| LOQ (ppb) | High | 54.2 | 34.0 | 43.7 | 5.4 | 73.1 | 67.5 |
|  | Low | 9 | - | 4 | 9 | - | 16 |
| Repeatability (%) | High | 0.9-2.0 | 0.6-10.3 | 0.7-11.5 | 1.5-13.1 | 0.4-4.3 | 1.6-4.0 |
|  | Low | 0.5-3.3 | - | 1.2-2.9 | 0.7-1.5 | - | 4.5-12.6 |
| Reproducibility (%) | High | 4.4 | 6.1 | 9.6 | 3.1 | 6.0 | 3.3 |
|  | Low | 1.5 | - | 2.7 | 4.1 | - | 5.5 |
| Recovery from standard addition (%) | High | 76.4 ± 6.0 | 69.8 ± 6.0 | 65.0 ± 8.9 | 0.0 | 38.4 ± 5.5 | 53.4 ± 4.4 |
|  | Medium | 76.7 ± 7.5 | 66.7 ± 4.7 | 60.9 ± 2.4 | 0.0 | 34.4 ± 8.3 | 50.4 ± 9.2 |
|  | Low | 56.9 ± 5.9 | 49.0 ± 7.3 | 59.2 ± 3.5 | 0.0 | 32.7 ± 2.8 | 40.9 ± 11.7 |
